# Turbocharging introgression breeding of perennial fruit crops: a case study on apple

**DOI:** 10.1038/s41438-020-0270-z

**Published:** 2020-04-01

**Authors:** Satish Kumar, Elena Hilario, Cecilia H. Deng, Claire Molloy

**Affiliations:** 1 The New Zealand Institute for Plant and Food Research Limited, Hawkes Bay Research Centre, Havelock North, New Zealand; 2 The New Zealand Institute for Plant and Food Research Limited, Mount Albert Research Centre, Auckland, New Zealand

**Keywords:** Genetics, Plant sciences

## Abstract

The allelic diversity of primitive germplasm of fruit crops provides a useful resource for introgressing novel genes to meet consumer preferences and environmental challenges. Pre-breeding facilitates the identification of novel genetic variation in the primitive germplasm and expedite its utilisation in cultivar breeding programmes. Several generations of pre-breeding could be required to minimise linkage drag from the donor parent and to maximise the genomic content of the recipient parent. In this study we investigated the potential of genomic selection (GS) as a tool for rapid background selection of parents for the successive generation. A diverse set of 274 accessions was genotyped using random-tag genotyping-by-sequencing, and phenotyped for eight fruit quality traits. The relationship between ‘own phenotypes’ of 274 accessions and their general combining ability (GCA) was also examined. Trait heritability influenced the strength of correspondence between own phenotype and the GCA. The average (across eight traits) accuracy of predicting own phenotype was 0.70, and the correlations between genomic-predicted own phenotype and GCA were similar to the observed correlations. Our results suggest that genome-assisted parental selection (GAPS) is a credible alternative to phenotypic parental selection, so could help reduce the generation interval to allow faster accumulation of favourable alleles from donor and recipient parents.

## Introduction

Growing demand for nutrient-rich foods, increasing biotic and abiotic risks in fruit production, and evolving consumer preferences require development of novel cultivars. The narrow genetic base of advanced genetic material generally inhibits introduction of new traits, hence utilising wild or semi-wild genepools as sources of genetic variation is a common breeding practice. Introduction of novel genes/traits from exotic germplasm have been achieved through two main strategies^1^: firstly, pre-breeding of primitive/exotic germplasm using recurrent selection, then using enriched germplasm as a source for crossing with elite lines; and secondly, backcrossing or pseudo-backcrossing (PBC) for introgression of known major genes from the donor into elite lines or commercial cultivars. The first approach increases the frequency of desirable genes and can help develop ‘pre-bridging’ germplasm, which can later be used as a pollen source for making crosses with elite lines^2^.

Pre-breeding of fruit crops germplasm using recurrent selection is very rare^3^, so most efforts have focussed on introgression of major genes (e.g. disease resistance) using a PBC strategy where foreground selection is carried out using marker assisted selection (MAS) and the resistant seedlings are then planted in the orchard for fruit quality and other trait evaluation^4,5^. PBC often results in ‘linkage drag’ (i.e. linkage between the gene of interest and undesirable genes that are inherited together from the donor parent), so repeated PBC with elite parents is applied to minimise the non-desirable genomic content of the donor parent in selected seedlings. In addition to the foreground MAS for major genes, a marker-based background selection (which involves selection of only those resistant seedlings that have inherited the least proportion of donor genome) can also be incorporated in a PBC breeding strategy^6–9^.

The breeding cycle length for outbred perennial fruit crops is long (for example, about 7 years for apple and pear), so pre-breeding generally takes decades to develop parents suitable for crossing with elite lines. Agro-technical (e.g. growth chamber) methods have been suggested to reduce the juvenility period^8,10^. Alternatively, a transgenic early flowering approach for the introgression of fire-blight resistance from the wild apple ‘Evereste’, showed that five breeding cycles could be completed within seven years^9^. However, transgenic plants are still regulated in many countries, so this strategy cannot be used for commercial cultivar breeding at the moment.

Genomic selection (GS) has been shown to be an effective tool to reduce the cycle length for pre-breeding^2,11,12^ and commercial cultivar breeding^13^. In GS-based parental breeding schemes, deleterious variation can be deselected to increase favourable allele frequencies irrespective of their origin, thus minimising linkage drag^2,12^. Numerous traits of interest could potentially be controlled by multiple genes, so GS would be ideal for introgressing traits of higher genetic complexity relatively faster than conventional breeding^14–16^.

Estimated breeding values (EBV) are commonly used to rank individuals in a breeding programme. An individual’s own phenotype (e.g. phenotypic records on a seedling) is one indicator of its EBV. Alternatively, EBV of an individual/seedling can be obtained from the performance of its progeny. In pre-breeding programmes of perennial fruit crops, own phenotype of seedlings is generally used for selection of parents for the next generation. However, own phenotype may not be a reliable indicator of EBV, especially for low-heritability traits, which could lead to sub-optimal parental selection^17^. Given the long time it takes to obtain progeny-based performance, fruit breeders continue with this current practice, accepting the known risks. Schlathölter et al.^9^ proposed using GS to identify parents for the next generation, but the efficacy of genome-assisted parental selection (GAPS) has not yet been fully established for pre-breeding programmes of perennial fruit crops. GAPS would help shorten the parental breeding cycle, so the main objectives of this study were to evaluate: (1) the correlation between seedlings own phenotype and progeny-test performance (i.e. general combining ability; GCA); (2) the accuracy of GAPS to predict own phenotype and GCA.

## Material and methods

### Plant material and phenotypes

A recurrent selection based pre-breeding programme was initiated during the early 1990s when about 35,000 apple seeds representing 500 open-pollinated families were imported from different countries into New Zealand^18^. An open-pollinated (OP) family represented seeds (which were pollinated by random pollen) harvested from a female parent. There was no passport data available on female accessions, and majority of these accessions belong to the domesticated species *Malus* × *domestica*. Seedlings were grown on their own roots, and after phenotypic evaluation for adaptation and fruit quality traits, a subset of c. 500 seedlings representing genetic diversity of the first-generation population, were identified. These 500 selections were propagated onto ‘M9’ rootstock and planted in duplicate at the Havelock North research orchard of Plant & Food Research for further assessment and long-term conservation. Nearly 350 (70%) of these accessions were used as parents to create a second generation, with each parent being involved, on average, in two crosses. The second generation comprised c. 10,000 seedlings; of these about 200 accessions (20%) were used as parents to create a third generation^3^. Outstanding accessions from this pre-breeding programme have been used in Plant & Food Research’s cultivar/parental development programmes.

A set of 274 accessions was chosen for this study, taking completeness of own phenotypic data as well as progeny-test data into consideration. Own phenotypes of each accession were recorded, and their GCA performance was obtained based on the phenotypes of their progenies. Because of the time it takes for progeny testing, own phenotypes and progeny performance were observed over different years. Six fruits from each plant were assessed over two consecutive years. Fruit were harvested when judged mature based on a change in skin background colour, and when the starch pattern index was between 2 and 3 using a scale from 0 (full starch) to 7 (no starch). Six fruits from each plant were harvested each fruiting season over 2 consecutive years, and were stored for 42 days at 0.5 °C, then a further 1 day at 20 °C before evaluation. The phenotypes used for this study included fruit weight (AVFW; g), russet coverage (RUSS; 0–9 scale), fruit firmness (FIRM; kg/cm^3^), soluble solids content (SSC; %), crispness (CRISP; 0–9 scale), juiciness (JUIC; 0–9 scale), astringency (ASTR; 0–9 scale) and titratable acidity (TA; percentage of malic acid in fruit juice). The average value of six fruits were used to represent each phenotype of each accession. Further details of harvesting and assessment protocol were reported previously^3^.

### DNA extraction

Total genomic DNA was extracted from 274 accessions using the Qiagen DNeasy Plant mini kit with the following modifications: Three leaf discs (diameter 6 mm) were obtained with a hand-held 1 Hole Plier Punch sterilised by dipping in ethanol for about 1 min, followed by distilled water for ~15 s, then dried out with lint-free tissues. The leaf discs were placed in a 1.5-mL micro-centrifuge tube containing lysis buffer and RNase A. The leaf sample was ground by hand with a sterile plastic pestle until most of the tissue was disintegrated. The sample was processed as instructed by the vendor, except that the final elution of DNA was done twice, with 1 h of incubation for each elution event. Both eluates were combined and quality assessed on a Fragment Analyzer (Genomic DNA High Sensitivity kit). DNA preps with a Genomic DNA Quality number equal or larger than 6.4 were considered acceptable (threshold value set to 10,000 bp). The average concentration was 15 ng/µL (1.4 ng/µL minimum, 63.2 ng/µL maximum).

### Random tagged genotyping-by-sequencing library preparation

As a test to verify that the observed range of DNA concentration would produce random-tagged DNA for library preparation, a set of 64 preps representing such range was amplified by PEP-PCR (Primer extension pre-amplification) and enriched by TD-PCR (touch down) as described by Hilario et al.^19^. After confirming that enough amplicon template could be obtained from this range of DNA concentrations, all DNA samples were processed and random tagged genotyping-by-sequencing (rtGBS) libraries prepared using the restriction enzyme BamHI. This enzyme was chosen after screening 10 restriction enzymes bioinformatically and later experimentally, according to the calculation sheet provided by the Gene Pool at the University of Edinburgh (http://genomics.ed.ac.uk/) by Mark Blaxter’s group, and uploaded in protocols.io (https://www.protocols.io/view/genotyping-by-sequencing-gbs-library-protocols-kzmcx46/abstract).

Two quality control steps were implemented: the first after random-tagging the DNA, and the second after amplifying the rtGBS library. Any samples that did not produce a library were repeated. Once all samples produced a library, five pools were prepared by combining a maximum of 56 libraries per pool. The pools were concentrated and quantified by fluorescence before and after size selection using a 3% agarose gel (250–650 bp). The sized fragments were assessed by gel electrophoresis, quantified by fluorescence and sent to Australian Genome Research Facility (AGRF), Brisbane, for sequencing. Each pool of rtGBS libraries was sequenced in one lane, on the Illumina HiSeq2000 platform, in single-end mode.

### Sequencing data analysis and variant calling

Sequencing data were downloaded from AGRF and data integrity confirmed with md5sum. Data quality was checked with FastQC-0.11.2. Barcode ligation proficiency and enzyme digestion efficiency were verified with fastq-multx in ea-utils.1.1.2-537 package in two steps: sample barcodes (sequence trimmed after de-multiplexing) and BamHI residue sequence (not-trimmed), both with a minimum phred score of Q20. For genotyping, we used the TASSEL3 pipeline^20^. First of all, 64-base long unique tags were extracted from the compressed fastq file for each pool through ‘FastqToTagCountPlugin’ with a minimum count of 3. Tags from the five pools were merged with ‘MergeMultipleTagCountPlugin’ to create the overall tag count table, then converted to Fastq format with ‘TagCountToFastqPlugin’. Afterwards tag sequences were mapped to Malus_x_domestica.v1.0-primary genome^21^ using Bowtie2^22^ with parameters ‘--very- sensitive --end-to-end’ in multi-thread mode. The Sequence Alignment Map (SAM) file was converted to Tags on Physical Map (topm) format using ‘SAMConverterPlugin’ followed by ‘ModifyTBTHDF5Plugin’ to achieve HDF5 format for rapid accessing of large datasets. In the meantime, Tags by Taxa (TBT) data in HDF5 format were created from all the raw sequencing files together with the tag count table using the SeqToTBTHDF5Plugin. To speed up variant calling, we ran the TagsToSNPByAlignmentPlugin for each pool against each *Malus* chromosome in parallel. Single nucleotide polymorphisms (SNPs) called on the same chromosome from different pools were merged with MergeDuplicateSNPsPlugin, while duplicate samples were combined with MergeIdenticalTaxaPlugin. The combined SNPs were filtered using GBSHapMapFiltersPlugin with a minimum site coverage (-mnScov) 0.9, minimum taxa coverage (-mnTCov) 0.8 and minimum minor allele frequency (-mnMAF) 0.05. The missing SNP genotypes were imputed using BEAGLE^23^ through the R package Synbreed^24^.

### Analysis of own phenotypes

As the measurements of own phenotypes of 274 accessions were repeated over two successive years, the following individual-tree mixed linear model (MLM) accounting for permanent environment effect was used for each trait:^13,25^

1 $$y = Xb + Za + Zp + e$$

where ***y*** is the vector of observations, ***b*** is the vector of fixed effects (overall mean, harvest year); ***a, p*** and ***e*** are vectors of random additive genetic effects, permanent environment effects, and residual effects, respectively. The matrix ***X*** is the incidence matrix for the fixed effects and ***Z*** is the incidence matrix relating observations to seedlings. The associated variances with the random effects, ***a***, ***p*** and ***e*** were $\sigma _a^2$, $\sigma _p^2$ and $\sigma _e^2$, respectively. Pedigree records were used to derive additive genetic relationships among all 274 accessions, and then ASReml software^26^ was used to obtain best linear unbiased prediction of additive effects (BLUP-BV) of each accession for each trait. These BLUP-BVs were later used for GS model development.

### Analysis of progeny test data

For each trait, the GCA of all 274 accessions was obtained from phenotypes of their offspring. Individual-tree MLM (Eq. 1) was used for this analysis, and additive genetic relationships among offspring, derived from their pedigree records, were taken into account. Estimates of narrow-sense heritability (*h*^2^) of each trait were obtained as the ratios of additive variance $\left( {\sigma _a^2} \right)$ to the total phenotypic variance $(={\sigma _a^2} + {\sigma _p^2} + {\sigma _e^2})$. The correspondence between BLUP-BV (derived from own phenotype) and GCA (derived from progeny phenotypes) was estimated as the product-moment correlation between these two measures.

### Genomic prediction and validation

BLUP-BV, obtained from Eq. 1 using own phenotypes of 274 accessions, were used as ‘phenotypes’ for the development and validation of genomic predictions as:

2 $$y = \mu {\mathbf{1}}_{\mathbf{n}} + Zg + \varepsilon$$

where ***y*** is a vector (274 × 1) of BLUP-BVs for a given trait; *μ* is an intercept, **1**_**n**_ is a vector of 1 s; ***Z*** is a design matrix relating observations to accessions, ***g*** is a vector of genomic breeding values with a normal distribution; ***g*** ~ *N*(0, ***G***$\sigma _a^2$), where ***G*** is a realised genomic relationship matrix derived using genome-wide SNPs^27^.

For each trait, the accuracy of genomic prediction was assessed through an 11-fold cross-validation. Records were randomly partitioned into 11 subsets of 25 records each (except one subset with 24 records). The records in one subset were in-turn set to missing values and were predicted using the model developed from the remaining 10 subsets, until phenotypes in all subsets were predicted. This 11-fold cross-validation scheme was repeated 20 times, and the prediction accuracy was calculated as the correlation between observed BLUP-BV and predicted genomic breeding value (GEBV) in the validation set. In addition, correlations between GEBV and GCA were also obtained in each validation set. Equation 2 was implemented using R package BGLR^28^.

## Results

About 5000 offspring (derived from cross-pollination) and 274 parents were phenotyped: the distribution of phenotypes in these two groups is presented in Supplementary Fig. S1. The average fruit weight (AVFW) in parental population was higher than in the progeny population (136 versus 112 g), and fruit firmness (FIRM), soluble solids content (measured as °Brix) and titratable acidity (TA) were slightly higher in the parental population (Supplementary Fig. S1; Table 1). The average values of all other traits were similar in both populations. Except for AVFW, phenotypic variability was similar or higher in the progeny population than in the parental phenotypes (Table 1).

**Table 1:** The overall average and standard deviation (SD) of various phenotypes (AVFW, RUSS, AVFF, SSC, CRISP, JUICE, ASTR, TA) of parental and progeny populations

	Own phenotype		Progeny phenotype	
Trait (unit)	Average	SD	Average	SD
AVFW (g)	136	90	112	65
RUSS (0–9)	2.5	0.99	2.4	0.96
AVFF (kg/cm^3^)	7.8	2.5	6.8	2.3
SSC (°Brix)	15.4	1.8	14.2	2.1
CRISP (0–9)	3.5	1	3.3	1.2
JUIC (0–9)	2.6	1.2	3.1	1.4
ASTR (0–9)	0.9	1.1	1	1.2
TA (%)	0.83	0.37	0.72	0.51

*AVFW* fruit weight, *RUSS* russet, *AVFF* fruit firmness, *SSC* soluble solids content, *CRISP* crispness, *JUICE* juiciness, *ASTR* astringency, *TA* titratable acidity

**Fig. 1:**
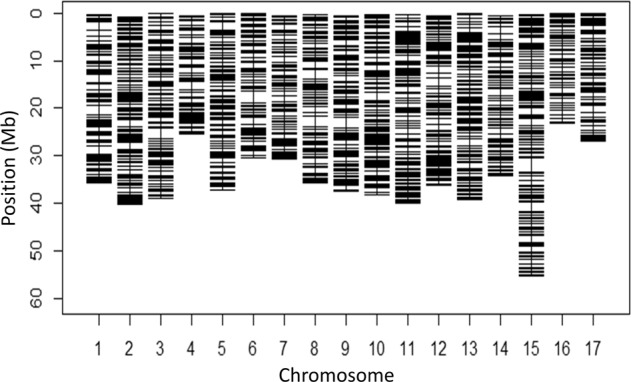
Distribution of the final set of over 6000 SNPs across 17 chromosomes of apple genome

**Fig. 2:**
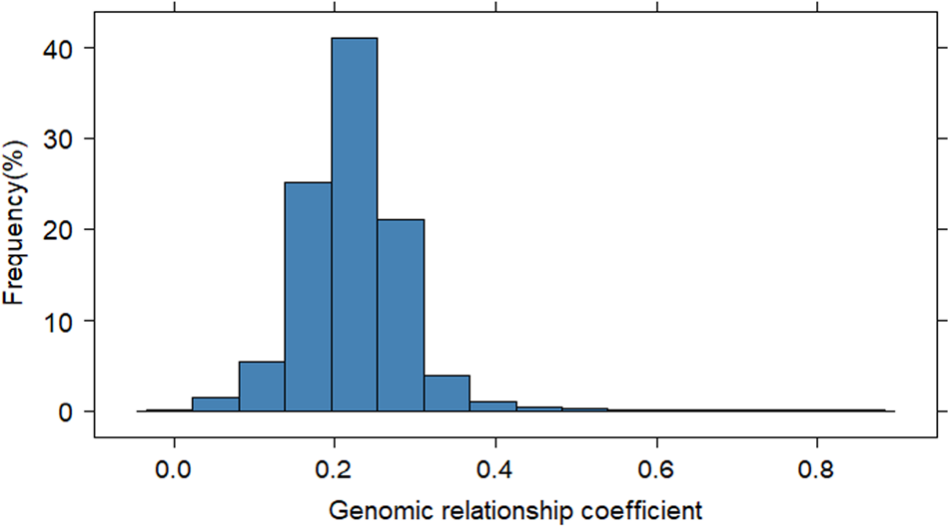
Distribution of genomic coefficient of relationships among 274 apple accessions

Initially, about 50 K SNPs were obtained following the filtering criteria described in the Methods section. SNPs were subsequently discarded using these criteria: more than 10% missing data; minor allele frequency (MAF) < 5%; and SNPs separated by one bp. As a result, a total of about 6400 high-quality SNPs, varying from 246 SNPs on LG16 to 594 SNPs on LG15, were retained (Fig. 1). Overall the final missing data rate was about 4%, and the missing SNP genotypes were imputed before further analyses. Using these SNPs, the pair-wise genomic relationship among our 274 accessions varied between 0 and 0.7 with an average relationship coefficient of 0.22 (Fig. 2). Within-accession heterozygosity (the proportion of SNP loci that were heterozygous) ranged from 0.11 to 0.44 among 274 accessions, with an average of 0.23.

**Fig. 3:**
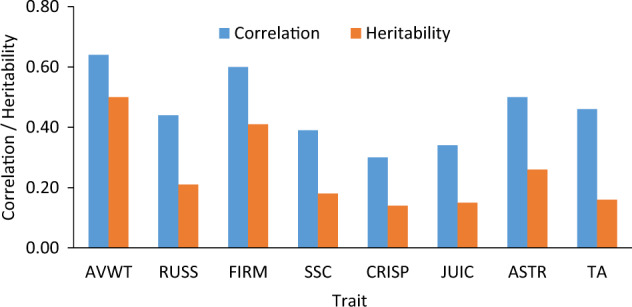
Correlation between own phenotype and progeny-based performance, and narrow-sense heritability for various apple fruit traits (AVWT fruit weight, RUSS russet, FIRM firmness, SSC soluble solids content, CRISP crispness, JUIC juiciness, ASTR astringency, TA titratable acidity)

Narrow-sense heritability (*h*^2^), based on progeny test data, varied from 0.15 (for CRISP and JUIC) to 0.50 (AVFW). After AVFW, fruit firmness was the second most heritable (0.41) trait, followed by ASTR (0.26) and RUSS (0.21) (Fig. 3). Estimated correlation between own performance (BLUP-BV) and progeny-based performance (GCA) was 0.60 or higher for AVWT and FIRM, and varied between 0.30 and 0.50 for all other traits (Fig. 3). Further investigation of data presented in Fig. 3 showed that, across all traits, the relationship between own phenotype and GCA was highly correlated (0.94) with the square-root of trait heritability.

**Fig. 4:**
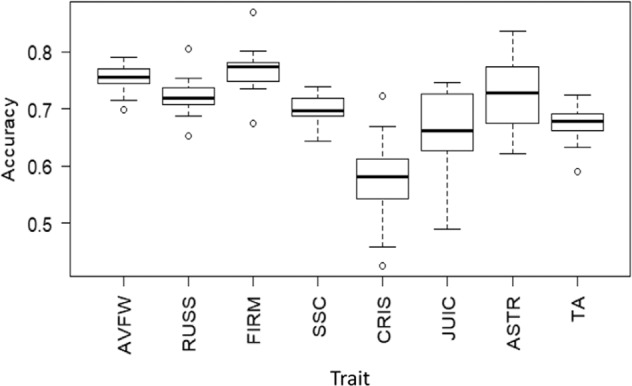
Genomic predicted accuracy of own phenotypes, and correlation between predicted/realised own phenotype and general combining ability (GCA) for various apple fruit traits (AVWT fruit weight, RUSS russet, FIRM firmness, SSC soluble solids content, CRISP crispness, JUIC juiciness, ASTR astringency, TA titratable acidity)

Accuracy of genomic predictions of own phenotype (i.e. correlation between GEBV and BLUP-BV) is shown in Fig. 4. The average prediction accuracy was higher than 0.70 for AVWT, RUSS, FIRM and ASTR; and varied between 0.60 and 0.70 for CRISP, JUIC, SSC and TA (Fig. 4). Small circles outside a box-and-whisker plot represent those values which is less than first-quartile (Q1) or greater than third-quartile (Q3) by more than 1.5 times the interquartile range (Q3–Q1).

**Fig. 5:**
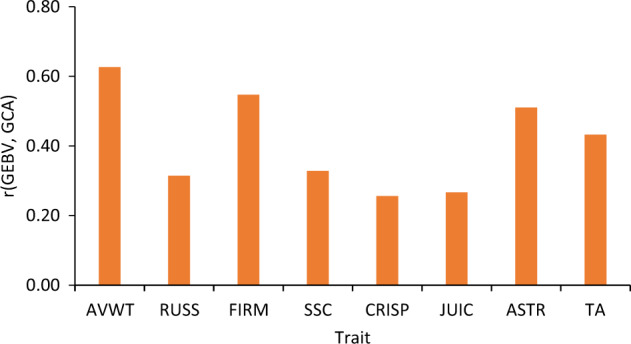
Correlation between own predicted performance (GEBV) and general combining ability (GCA) for various apple fruit traits (AVWT fruit weight, RUSS russet, FIRM firmness, SSC soluble solids content, CRISP crispness, JUIC juiciness, ASTR astringency, TA titratable acidity)

The average correlation between predicted own performance (GEBV) and GCA was higher than 0.50 for AVWT, FIRM and ASTR; and between 0.30 and 0.50 for RUSS, SSC and TA; and between 0.25 and 0.30 for CRISP and JUIC (Fig. 5), very similar to the correlation between observed performance (BLUP-BV) and GCA (Fig. 3).

## Discussion

A key goal of creating pre-bridging germplasm is to evaluate and exploit the available genetic variability using recurrent selection cycles, to generate advanced genetic material suitable as parents for cultivar development populations^2^. In contrast, the focus of traditional PBC schemes is to introduce monogenic traits such as disease resistances from donor accessions, and to reduce the unwanted share of wild/primitive genome through multiple PBC cycles. Phenotypic screening for background selection can be slow and expensive. To fast-track introgression, MAS for the donor gene (i.e. foreground selection) could be followed by marker-based background selection to identify seedlings with high genomic similarity to the elite lines^6–8,29^.

Genomic-similarity based selection could ensure the high recovery of genomic content of the recipient lines when using a high-density genotyping platform. However, in most pre-breeding programmes, the major goal is not necessarily to capture the recipient’s genomic background, but to build on some specific economically important traits. High genomic similarity may not necessarily deliver high phenotypic similarity to the elite parent, hence GS models, derived using genotype and phenotypes, would provide a more efficient and targeted approach for background selection. Thus, GS would lead to desired genetic improvement of target traits relatively faster than using genomic-similarity based selection^30^.

GS would be a more efficient strategy for background selection, especially for complex traits, as favourable alleles from the donor and recipient lines can be targeted across the whole genome. Accuracy of GS studies in apple and pear cultivar breeding were encouraging^13,31–33^, but the current applications of GS in perennial fruit crops are generally aimed at skipping Stage 1 seedling evaluation so that candidates for Stage 2 evaluation can be identified based on GEBV—hence reducing the cultivar development timeline by at least five years^13^. Although, the candidates selected for Stage 2 testing could also be used as parents of the next generation, it is suspected that their worthiness as parents could be compromised mainly because of recombination events.

The results that relationship between own phenotype and GCA was highly correlated with the square-root of trait heritability, suggested that heritability expresses the reliability of phenotypic value as a guide to the breeding value. For this reason, the determination of heritability of breeding target traits is one of the first objectives in breeding programmes. The accuracy of predicting phenotypes of our 274 accessions was 0.58 for the least heritable trait (CRISP) and 0.75 for the most heritable trait (AVWT). Trait heritability, genetic architecture of the trait, and genetic relatedness between the training and validation sets, are among the key factors influencing the accuracy of genomic predictions^34–36^. Relatively smaller training population sizes and lower average pairwise genomic relatedness would have contributed to lower prediction accuracies than those reported earlier for the same traits^13^.

This study showed that the degree of correspondence between own phenotype and progeny-based performance (i.e. GCA) was strongly influenced by the heritability i.e. for low-heritability traits, own phenotype could be a poor indicator of GCA^25^. Nevertheless, pre-breeding programmes of perennial fruit crops (e.g. apple and pear) use seedlings’ own phenotypes for background selection of parents of the next generation. Our results suggest that parental selection based on genomic prediction of own phenotypes would be similarly accurate as this conventional selection. Hence, GS approach would obviate the need for costly and time-consuming phenotypic screening for background selection in pre-breeding programmes. Linkage disequilibrium between pairs of loci would decay over successive backcross cycles, which could require recalibration of GS model every couple of generations.

A combination of genome editing (GE) techniques (e.g. CRISPR-Cas) and GS has been shown to significantly enhance the response to selection in animal breeding schemes^37^, and a GE-GS combination was also proposed for pre-breeding schemes to develop climate-smart crops^38^. GE is still a controversial technique, so for the foreseeable future GS-based pre-breeding schemes could improve efficiency in introgressing favourable polygenic traits of donors into high-quality recipient lines whilst also minimizing linkage drag^2,11^.

In parental breeding programmes of various fruit crops, MAS is commonly used to screen seedlings for monogenic traits (e.g. disease resistance) and then selection for fruit quality traits is carried out based on field phenotypes. Results from this study showed that GS is a credible alternative to phenotypic selection, so for parental breeding of perennial fruit crops we suggest a two-stage selection might be employed—where MAS is used for foreground selection for monogenic traits and GS is used for background selection for fast recovery of the best genomic content of the donor and recipient parents. Prospective parents selected based on MAS and GS could then be propagated in fast-growth chambers to facilitate early transition from the juvenile to the adult phase. Such turbocharged regimes could reduce the breeding cycle of some fruit crops (e.g. apple, pear) to two years, compared with the seven years of a conventional scheme.

## Supplementary information

Supplementary Fig S1
