# Supplementary material for: Turbocharging introgression breeding of perennial fruit crops: a case study on apple
Source: Hortic Res. 2020 Apr 1;7:47. doi: 10.1038/s41438-020-0270-z (PMC7109137; doi:10.1038/s41438-020-0270-z)
Supplement: Supplementary file 1 — Supplementary Fig S1 [file 41438_2020_270_MOESM1_ESM.docx]

**Supplementary Fig. S1**. Phenotypic distributions of apple parental and progeny populations.
